# Sentinel-2 and Unmanned Aerial Vehicle (UAV) Imagery for Irrigation Scheduling in Fodder Maize: A Comparative Remote Sensing Approach

**DOI:** 10.3390/plants15152265

**Published:** 2026-07-24

**Authors:** Nuria Aide López Hernández, Victor Manuel Rodríguez Moreno, Ricardo Israel Ramírez Gottfried, Ramón Trucíos Caciano, Marco Antonio Inzunza Ibarra, Aldo Rafael Martínez Sifuentes

**Affiliations:** 1 National Center for Disciplinary Research in the Relationship Water, Soil, Plant, and Atmosphere, National Institute of Agricultural and Livestock Forestry Research, Km. 6.5 Margen Derecha Canal de Sacramento, Gomez Palacio 35079, Mexico; inzunza.marco@inifap.gob.mx; 2 National Laboratory of Modeling and Remote Sensing, National Institute for Forestry, Agriculture and Livestock Research, North Central Regional Research Center, Experimental Field Station “Pabellón”, Highway Aguascalientes-Zacatecas km 32.7, Pabellón de Arteaga 20670, Mexico; rodriguez.victor@inifap.gob.mx; 3 Laguna Unit, Antonio Narro Autonomous Agrarian University, Periférico Raúl López Sánchez s/n, Col. Valle Verde, Torreon 27054, Mexico; gottfried_ricardo@hotmail.com; 4 North Central Regional Research Center, National Institute of Agricultural and Livestock Forestry Research, Blvd. José Santos Valdés 1200 West, Matamoros 27440, Mexico; trucios.ramon@inifap.gob.mx

**Keywords:** crop coefficient, crop water requirements, evapotranspiration estimation, irrigation management, precision agriculture

## Abstract

Accurate estimation of crop water requirements is essential to improve irrigation efficiency for forage maize production. This study compared satellite- and UAV-derived normalized difference vegetation index (NDVI) models for estimating crop coefficients (K_c_) and evaluated their operational performance for irrigation scheduling. K_c_–NDVI models were developed during the 2023 growing season and subsequently validated under field conditions during the 2024 season in two forage maize hybrids (N83N5 and Matador) under three irrigation strategies: conventional producer irrigation (ID1), satellite-based irrigation scheduling (ID2), and UAV-based irrigation scheduling (ID3). Both NDVI sources exhibited strong relationships with K_c_, with higher calibration accuracy for the UAV model (R^2^ = 0.9414) than for the satellite model (R^2^ = 0.8278). The UAV-based model applied 23–30% less irrigation water, maintaining high water productivity but also reducing crop growth, forage yield, and nutritional quality. In contrast, satellite-based irrigation scheduling promoted greater crop growth and produced the highest forage yield, reaching 59.8 t ha^−1^ in hybrid N83N5 while maintaining efficient water use. This treatment also improved forage quality by increasing dry matter and starch concentrations while reducing fiber fractions. The findings highlight the complementary potential of satellite and UAV imagery in precision irrigation and underscore the trade-offs between spatial detail, temporal resolution, and operational scalability. Furthermore, the results demonstrate that a stronger K_c_–NDVI relationship does not necessarily translate into improved irrigation scheduling performance. Under the conditions evaluated, the satellite-based model provided the best balance between water use, forage yield, and nutritional quality.

## 1. Introduction

Maize (*Zea mays* L.) is one of the most important forage crops worldwide and represents the main source of silage for dairy production systems, owing to its nutritional quality and high productivity [1,2]. However, in arid and semi-arid regions, such as Northern Mexico, irrigation is essential to achieve high biomass production, requiring more efficient irrigation management strategies [3]. Improving irrigation scheduling is therefore critical to maximize crop productivity while minimizing water consumption [4].

Crop evapotranspiration (ET_c_) is widely used to estimate crop water requirements and schedule irrigation. According to the FAO-56 methodology, ET_c_ is commonly calculated as the product of reference evapotranspiration (ET_0_) and K_c_ [5]. Therefore, accurate estimation of K_c_ is fundamental for reliable irrigation scheduling. Although FAO-56 provides generalized K_c_ values for maize, crop coefficients vary according to climate, management practices, cultivar characteristics, and local environmental conditions. Consequently, fixed K_c_ values may not accurately represent crop water use under specific production environments [6,7], motivating the development of site-specific approaches for K_c_ estimation.

Vegetation indices derived from remote sensing have emerged as practical tools for estimating K_c_. Among them, the NDVI has been extensively used because it is strongly related to canopy cover, leaf area development, biomass accumulation, and crop transpiration [8]. Several studies have reported strong relationships between NDVI and K_c_ in maize [9,10]. The most commonly used remote sensors for obtaining NDVI are satellites and unmanned aerial vehicles (UAVs). The Sentinel-2 Satellite platform has increasingly been used to derive NDVI-based K_c_ and evapotranspiration estimates in maize, providing a practical and cost-effective alternative for irrigation scheduling [11,12,13]. Moreover, recently, UAVs provide imagery with substantially higher spatial resolution than satellite systems, allowing detailed characterization of within-field variability. This approach provides site-specific estimates of crop evapotranspiration and supports precision irrigation management [10,14,15]. However, UAV-based monitoring generally requires greater operational effort and may be limited by flight logistics, weather conditions, and data-processing requirements [16,17].

Although numerous studies have developed K_c_–NDVI relationships using satellite or UAV imagery, most research has focused on model calibration and statistical performance. Consequently, the agronomic implications of using different remote sensing platforms for irrigation management remain insufficiently understood. Moreover, direct comparisons of their performance for developing irrigation-scheduling models under identical field conditions are still limited. This knowledge gap hinders the identification of the most appropriate remote sensing platform for operational irrigation management and highlights the need for studies evaluating both approaches within the same production environment. Therefore, the objective of this study was to evaluate the performance of satellite- and UAV-derived K_c_–NDVI models for irrigation scheduling in forage maize and to determine their effects on irrigation water requirements, crop growth, forage yield, and nutritional quality.

## 2. Results

### 2.1. K_c_–NDVI Linear Regression Models

**Figure 1:**
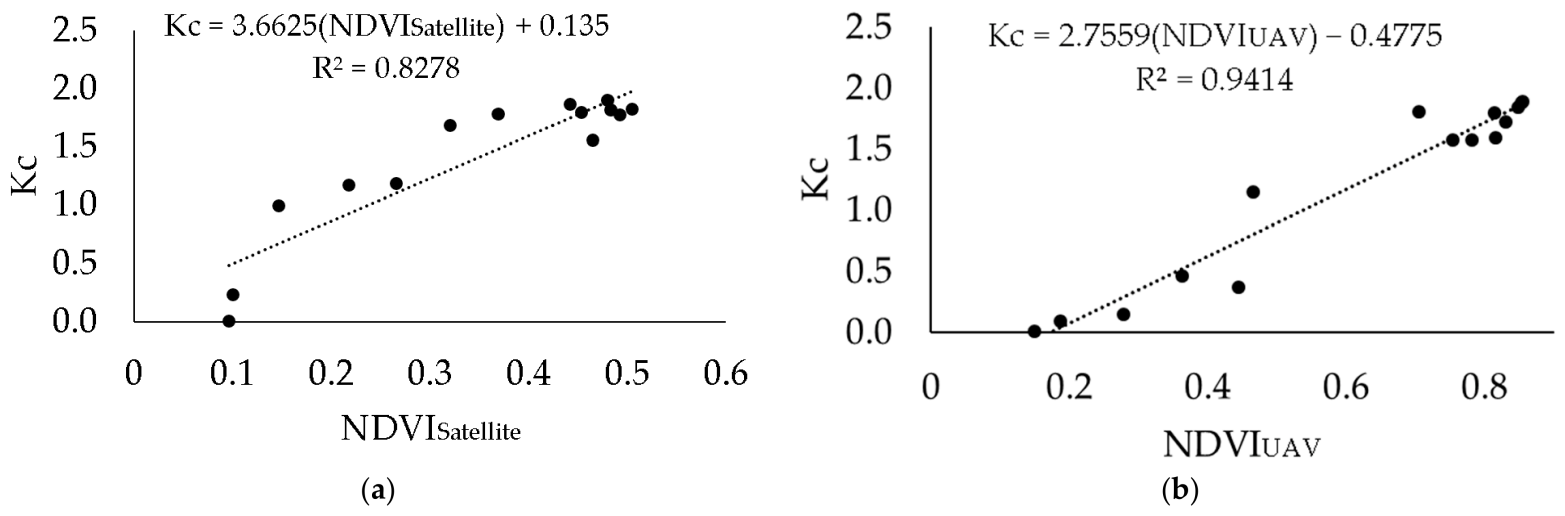
Linear relationships between remotely sensed NDVI and crop coefficient (K_c_) for fodder maize derived from (**a**) Sentinel-2 imagery (Kc–NDVI _Satellite_) and (**b**) UAV imagery (K_c_–NDVI _UAV_). The black circles indicate the observed data, and the dashed lines indicate the fitted linear regression models.

The linear regression models obtained between NDVI and K_c_ for both remote sensing platforms are presented in Figure 1. Both models exhibited positive linear relationships between NDVI and K_c_, indicating that increases in NDVI were associated with higher K_c_ values throughout fodder maize development. The regression models showed good fitting performance, demonstrating the capability of both Sentinel-2 and UAV-derived NDVI data to estimate K_c_ variability under field conditions. However, the UAV-based model exhibited a stronger relationship (R^2^ = 0.9414) compared to the satellite-based model (R^2^ = 0.8278), indicating greater sensitivity of high-resolution UAV imagery to canopy changes and crop vigor. Differences in the regression slopes and intercepts further indicate variations in the response of each sensing platform to canopy characteristics and spatial crop variability. Overall, the results demonstrate the strong potential of remotely sensed NDVI as a reliable predictor of K_c_ in fodder maize.

**Figure 2:**
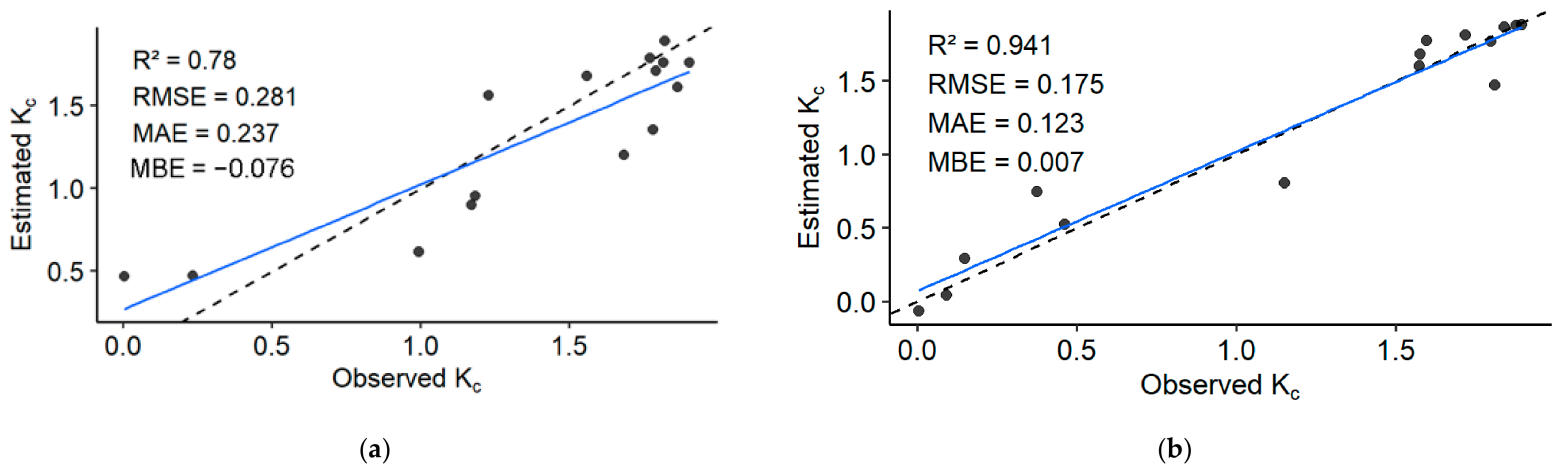
Performance comparison of Sentinel-2 (**a**) and UAV-based (**b**) models for crop coefficient (Kc) estimation in fodder maize. Black circles represent the observed and estimated Kc data pairs. The dashed 1:1 line represents perfect agreement between observed and estimated values, whereas the solid blue line corresponds to the fitted linear regression.

The goodness-of-fit of the regression models is presented in Figure 2 and indicates good agreement between observed and estimated K_c_ values for both regression models. The UAV-based model produced estimates that were more closely distributed around the 1:1 line than those from the Sentinel-2 model, reflecting its higher R^2^ and lower prediction errors. The goodness-of-fit statistics confirmed this pattern, with the UAV model exhibiting lower RMSE and MAE values and a mean bias error (MBE) close to zero, indicating greater accuracy and negligible systematic bias compared with the satellite-based model.

### 2.2. Irrigation Water Requirements

Cumulative irrigation depth differed among irrigation strategies and hybrids (Table 1). ID3 consistently applied the lowest irrigation depths in both hybrids, reducing total applied water by approximately 23% in N83N5 and 30% in Matador relative to ID1. In contrast, ID2 produced irrigation depths comparable to those of ID1, although a slight reduction was observed in N83N5.

### 2.3. In-Field Measurements

#### 2.3.1. Analysis of Variance of Mixed-Effects Models

Mixed-model analysis of variance (ANOVA) indicated that irrigation treatment and sampling time significantly affected stem diameter, plant height, SPAD (Soil Plant Analysis Development) chlorophyll index, and in situ NDVI throughout the crop cycle (*p* < 0.05). In contrast, hybrid effects were generally not significant, except for plant height, where the hybrid × time interaction was significant (*p* = 0.0162), indicating differences in growth dynamics between hybrids over time. Irrigation × time interaction was also significant for plant height (*p* = 0.0026), suggesting that irrigation effects varied across developmental stages. No significant triple interactions were detected for any variable (*p* > 0.05), indicating consistent responses among hybrids and irrigation treatments over time (Table 2).

#### 2.3.2. Stem Diameter

**Figure 3:**
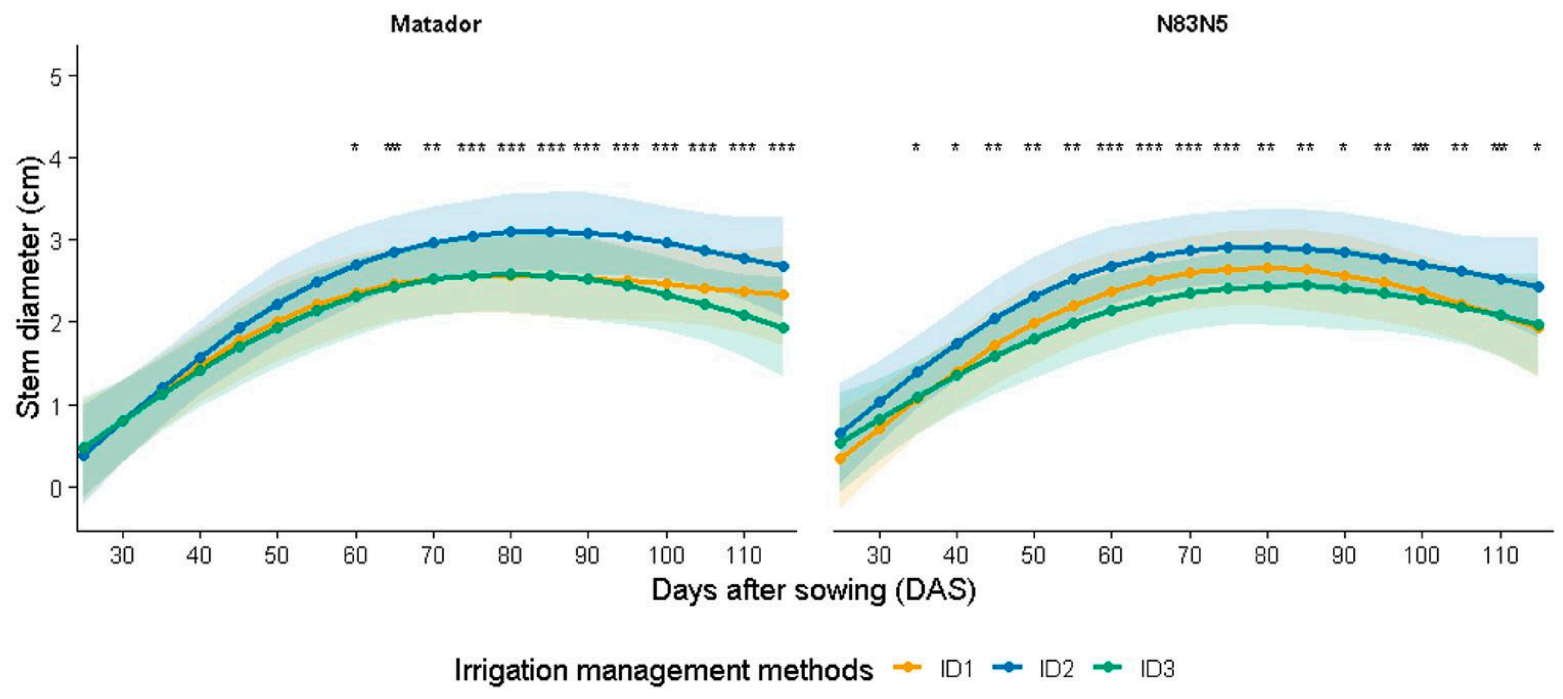
Stem diameter across days after sowing (DAS) under three irrigation strategies: traditional producer irrigation (ID1), satellite-based K_c_ irrigation scheduling (ID2), and UAV-based K_c_ irrigation scheduling (ID3). Solid lines represent estimated marginal means obtained from the mixed-effects model with CAR(1) covariance structure and shaded areas indicate 95% confidence intervals. Asterisks indicate significant differences among irrigation treatments according to Tukey’s test (* *p* ≤ 0.05, ** *p* ≤ 0.01, *** *p* ≤ 0.001).

The fitted curves showed a rapid increase in stem diameter during vegetative growth, reaching maximum values at approximately 80–90 DAS, followed by stabilization around tasseling and a slight decline toward physiological maturity (Figure 3). Across both hybrids, the ID2 treatment generally maintained the largest stem diameters, particularly during periods of high water demand (reproductive development), whereas ID3 produced the lowest values, under reduced irrigation depths. Nevertheless, reductions in stem diameter under ID3 remained relatively moderate, suggesting a tradeoff between water savings and vegetative development.

In Matador, significant differences between ID2 and the other irrigation treatments became evident after 60 DAS and were most pronounced during the reproductive stage (95–115 DAS). Compared with ID3, ID2 increased stem diameter by 0.38 to 0.75 cm, whereas differences relative to ID1 ranged from 0.36 to 0.55 cm. For N83N5, significant differences between ID2 and ID3 appeared earlier, at approximately 40 DAS, and remaining until the end of the season. Differences between these treatments ranged from 0.38 to 0.55 cm. Significant differences between ID1 and ID2 were less frequent and mainly occurred during late-season evaluations. In contrast, differences between ID1 and ID3 were generally limited in both hybrids. Detailed Tukey multiple comparison results are provided in Appendix A.1.

#### 2.3.3. Plant Height

**Figure 4:**
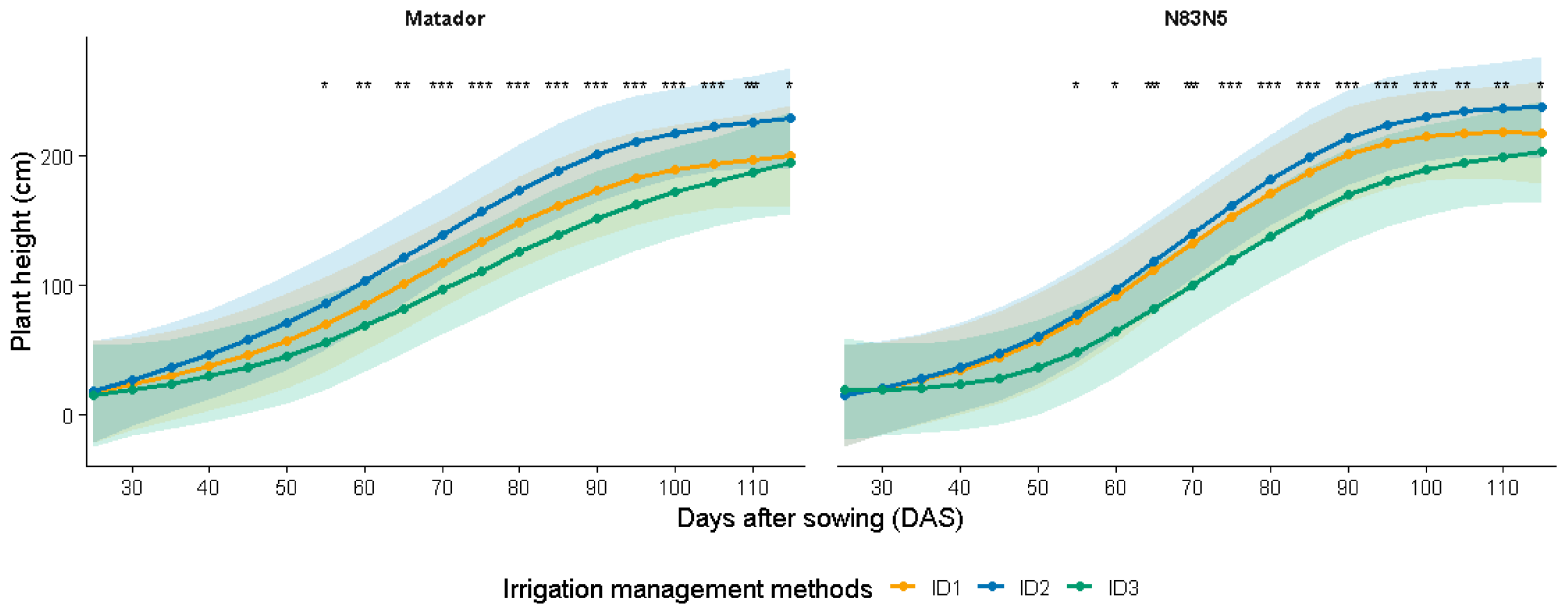
Plant height across days after sowing (DAS) under three irrigation strategies: traditional producer irrigation (ID1), satellite-based K_c_ irrigation scheduling (ID2), and UAV-based K_c_ irrigation scheduling (ID3). Solid lines represent estimated marginal means obtained from the mixed-effects model with CAR(1) covariance structure and shaded areas indicate 95% confidence intervals. Asterisks indicate significant differences among irrigation treatments according to Tukey’s test (* *p* ≤ 0.05, ** *p* ≤ 0.01, *** *p* ≤ 0.001).

Plant height increased progressively throughout the growing season in both hybrids, following a sigmoidal growth pattern (Figure 4).

Repeated-measures mixed models revealed that irrigation treatment effects depended on crop developmental stage, with significant differences among irrigation methods becoming evident mainly after 55 DAS. For both hybrids, ID2 consistently produced the greatest plant height values during most of the crop cycle, particularly throughout the rapid vegetative growth phase. In contrast, ID3 substantially reduced total applied water depth but also reduced plant growth, suggesting a stronger water limitation effect during vegetative development.

In Matador, significant differences were primarily detected between ID2 and ID3 from 55 to 115 DAS, with ID2 producing plants approximately 30 to 49 cm taller than ID3 during the period of maximum growth. Moreover, ID2 also exceeded ID1 between 95 and 110 DAS. For N83N5, ID2 similarly resulted in greater plant height than ID3 from 55 DAS onward, while ID1 showed intermediate performance and exceeded ID3 between 65 and 95 DAS. The absence of significant differences during early growth stages suggests that crop water demand was relatively low during initial vegetative development. However, as crop growth accelerated, differences among irrigation methods became progressively more pronounced. Complete Tukey multiple comparison results for all evaluation dates are presented in Appendix A.2.

#### 2.3.4. SPAD Chlorophyll Index

**Figure 5:**
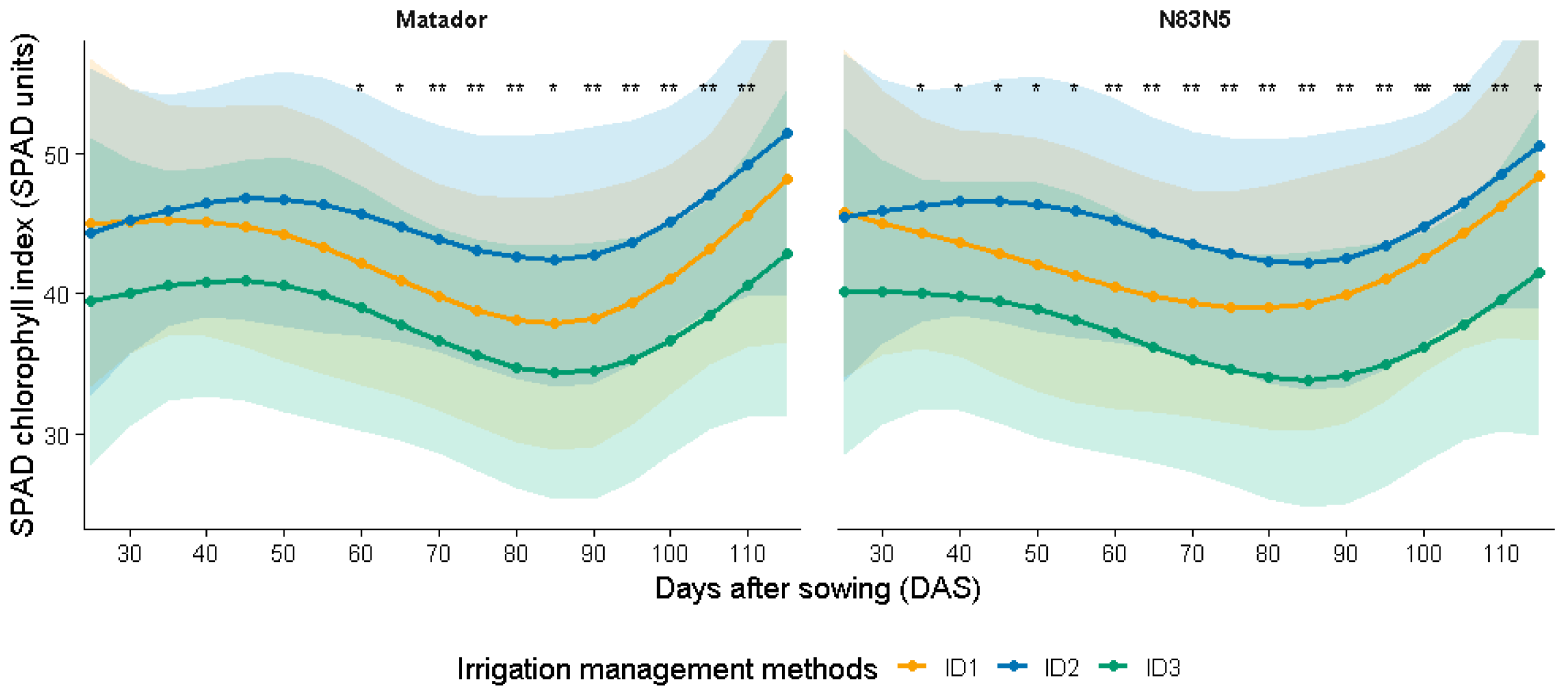
SPAD chlorophyll index across days after sowing (DAS) under three irrigation strategies: traditional producer irrigation (ID1), satellite-based K_c_ irrigation scheduling (ID2), and UAV-based K_c_ irrigation scheduling (ID3). Solid lines represent estimated marginal means obtained from the mixed-effects model with CAR(1) covariance structure and shaded areas indicate 95% confidence intervals. Asterisks indicate significant differences among irrigation treatments according to Tukey’s test (* *p* ≤ 0.05, ** *p* ≤ 0.01, *** *p* ≤ 0.001).

Figure 5 shows the fitted temporal trajectories of SPAD chlorophyll index for both hybrids under the three irrigation treatments. Temporal SPAD dynamics followed a similar pattern in both hybrids, characterized by relatively stable values during early vegetative growth, a decline during mid-season, and a gradual recovery toward physiological maturity. Across the growing season, the ID2 irrigation treatment consistently maintained the highest SPAD values, whereas the ID3 treatment showed the lowest values, particularly from mid-season onward, suggesting greater crop stress and reduced leaf chlorophyll status under lower water availability conditions. The ID1 treatment generally exhibited intermediate values between both irrigation strategies.

In Matador, significant differences between ID2 and ID3 became evident around 60 DAS and remained significant through late-season evaluations (*p* < 0.05). Differences between these treatments ranged from approximately 6.7 to 8.6 SPAD units, with the largest differences occurring during the reproductive stages. In contrast, no significant differences were detected between ID1 and the other irrigation treatments throughout most of the growing season. These results suggest that the ID2 irrigation strategy promoted greater chlorophyll retention and improved crop physiological status during periods of high water demand.

For N83N5, significant differences between ID2 and ID3 appeared earlier than in Matador, beginning at approximately 35 DAS and persisting until the end of the evaluation period. Differences between these treatments ranged from approximately 6.3 to 9.1 SPAD units throughout the season. Additionally, ID1 exhibited significantly greater SPAD values than ID3 at approximately 100 and 105 DAS, while no significant differences were detected between ID1 and ID2 during most evaluation dates. These results indicate that N83N5 responded earlier to differences in irrigation management compared with Matador. Detailed Tukey multiple comparison results for all evaluation dates are provided in Appendix A.3.

#### 2.3.5. In Situ NDVI

**Figure 6:**
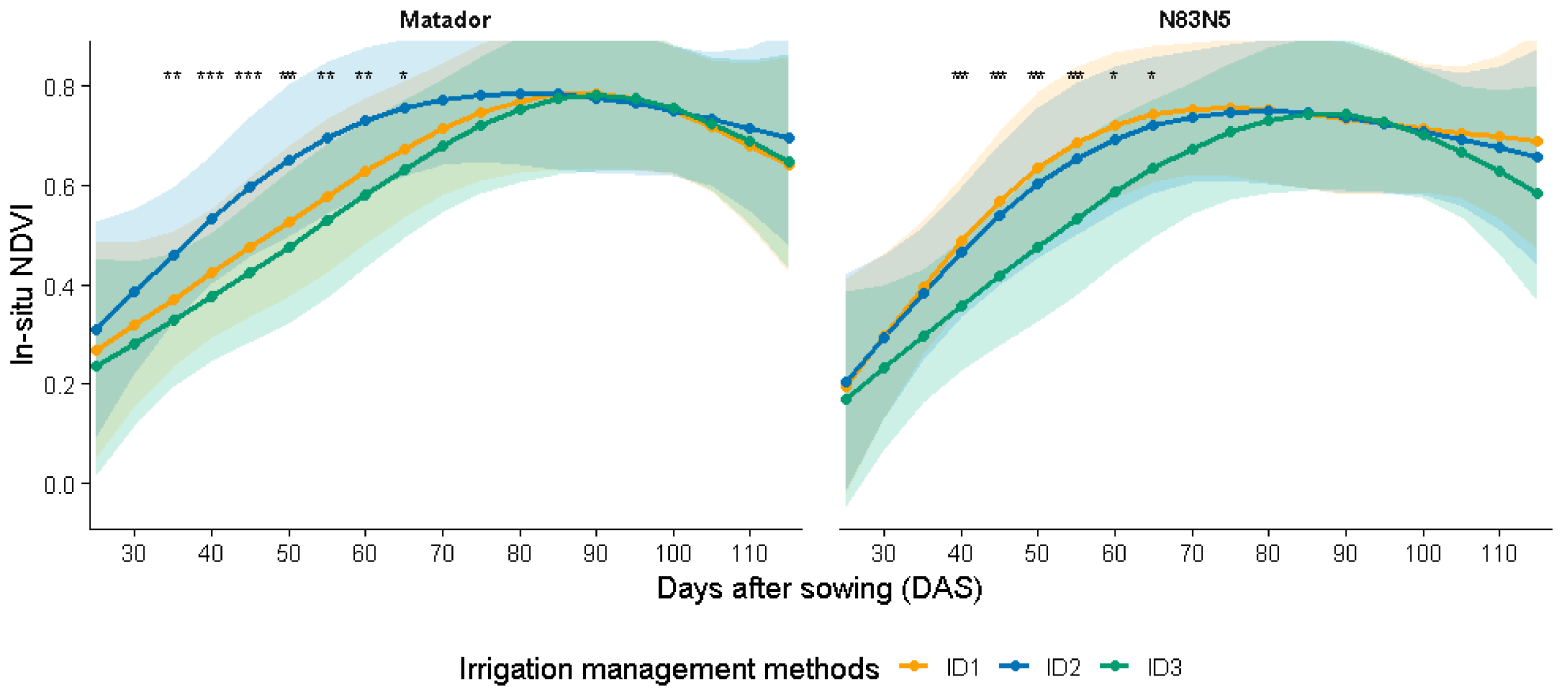
In situ NDVI during the crop cycle for two maize hybrids under three irrigation management methods: traditional producer irrigation (ID1), irrigation scheduling using the K_c_–NDVI_Satellite_ model (ID2), and irrigation scheduling using the K_c_–NDVI_UAV_ model (ID3). Solid lines represent estimated marginal means obtained from the mixed-effects model with CAR(1) covariance structure and shaded areas indicate 95% confidence intervals. Significant differences among irrigation treatments at each sampling date were identified using Tukey’s multiple comparison test (* *p* ≤ 0.05, ** *p* ≤ 0.01, *** *p* ≤ 0.001).

Figure 6 shows the fitted temporal trajectories of in situ NDVI measured with the GreenSeeker sensor for both hybrids under the three irrigation treatments. Across the growing season, NDVI values increased rapidly during vegetative growth, reached maximum values around 80–95 DAS, and gradually declined toward physiological maturity. In general, the ID2 irrigation strategy maintained the highest NDVI values during early and mid-season evaluations, whereas the ID3 treatment tended to show the lowest values, suggesting reduced canopy growth and greater crop stress under lower water availability conditions. The ID1 treatment generally exhibited intermediate responses.

In Matador, significant differences between ID2 and ID3 were detected from approximately 35 to 65 DAS (*p* < 0.05). During this period, NDVI values under ID2 exceeded those observed under ID3 by approximately 0.12 to 0.18 NDVI units. Significant differences between ID1 and ID2 were also detected between 40 and 50 DAS, with ID2 showing greater NDVI values than ID1. These results indicate that the ID2 irrigation strategy promoted earlier canopy development and greater vegetation vigor during the vegetative growth stage.

For N83N5, treatment differences followed a different pattern. Significant differences were primarily detected between ID3 and the other irrigation treatments from approximately 40 to 65 DAS. Compared with ID3, both ID1 and ID2 maintained greater NDVI values, with differences ranging from approximately 0.11 to 0.16 NDVI units. In contrast to Matador, no significant differences were detected between ID1 and ID2 during the evaluation period. These results suggest that reductions in irrigation depth under ID3 had a greater effect on early canopy development in N83N5.

Overall, temporal NDVI dynamics indicated that irrigation management influenced crop vigor and canopy development primarily during vegetative and early reproductive stages. Detailed Tukey multiple comparison results for all evaluation dates are provided in Appendix A.4.

### 2.4. Yield, Water Productivity and Bromatological Analyses

Yield, water productivity and forage quality parameters (Table 3) were significantly influenced by the interaction between maize hybrid and irrigation strategy.

The hybrid N83N5 under ID2 produced the highest forage yield (59.8 t ha^−1^), which was significantly greater than the remaining treatments. This treatment also showed the highest dry matter (DM) (40.55%) and starch concentration (37.88%), together with the lowest neutral detergent fiber (NDF) (37.90%), acid detergent fiber (ADF) (19.73%), and lignin contents (4.17%), indicating superior forage quality. In contrast, ID3 substantially reduced the applied irrigation depth in both hybrids, particularly in Matador (650 mm) and N83N5 (717 mm), representing water savings of approximately 30% and 23%, respectively, compared with ID1. Despite the lower irrigation depths, water productivity (WP) remained relatively high under ID3, reaching 6.49 and 6.09 kg m^−3^ in N83N5 and Matador, respectively. In N83N5, WP under ID3 was comparable to that obtained under ID2 (6.71 kg m^−3^), whereas in Matador it exceeded both ID1 and ID2.

Despite the reduction in irrigation water, ID3 was associated with increases in fiber fractions and lignin content. Both hybrids under ID3 exhibited the highest NDF and ADF values, as well as reduced starch concentrations, suggesting lower digestibility and energy content of the forage. The Matador hybrid generally showed lower forage quality compared with N83N5, particularly under ID3, where the lowest starch concentration (8.30%) and highest ADF values were observed. Crude protein content showed limited variation among treatments, ranging from 8.96 to 10.64%. Overall, ID2 improved forage productivity and nutritional quality while maintaining high WP, particularly in the N83N5 hybrid. In contrast, ID3 achieved similar or improved WP with lower irrigation inputs, although at the expense of some forage quality attributes.

## 3. Discussion

### 3.1. K_c_–NDVI Linear Regression Models

The relationship between NDVI and K_c_ in maize has been widely researched; however, most previous studies have relied on FAO-56 K_c_ values rather than direct field measurements. In this study, K_c_ was determined using a weighing lysimeter, providing a direct estimate of crop evapotranspiration under local conditions. Both satellite- and UAV-derived NDVI exhibited strong linear relationships with K_c_, confirming that NDVI effectively captured temporal variations in crop water use, with increasing NDVI values associated with higher evapotranspiration rates [18] and, consequently, greater K_c_ values [19,20]. Similar results have been reported using Landsat, MODIS, and UAV imagery, with coefficients of determination ranging from 0.79 to 0.97 [10,12,14,21,22,23].

The stronger relationship of the UAV-based model observed in this study may be partly attributed to its higher spatial resolution, which allows a more detailed characterization of canopy variability, thereby reducing the influence of mixed pixels and improving synchronization between spectral observations and crop development stages [16,24,25]. However, the differences between Sentinel-2 and UAV-derived models should not be attributed solely to spatial resolution. The two platforms also differ in spectral band configuration, image acquisition timing, radiometric calibration procedures, and data processing workflows. Furthermore, Sentinel-2 observations are additionally limited by fixed revisit intervals and atmospheric conditions. These factors may influence vegetation index retrieval and explain part of the discrepancies observed in K_c_–NDVI relationships [26,27,28]. Therefore, the contrasting K_c_–NDVI relationships observed in this study likely reflect the combined effects of multiple sensor-specific characteristics. In addition, although the models were calibrated during the 2023 growing season and applied under field conditions in 2024, both phases were conducted at the same experimental site. Consequently, the proposed approach has not yet been validated using an independent external dataset, and its applicability under different environmental conditions, management practices, or growing seasons remains to be confirmed through future studies.

### 3.2. Irrigation Water Requirements

Irrigation depths differed considerably among irrigation scheduling methods, particularly between ID2 and ID3. Although the UAV-derived K_c_–NDVI model showed a stronger statistical relationship with K_c_ than the satellite-based model, the UAV-based strategy consistently applied lower irrigation depths. Significant differences between ID2 and ID3 emerged after approximately 55 DAS in both hybrids, suggesting that the UAV-based model may have underestimated crop water requirements during the rapid canopy development [29]. In contrast, the satellite-based treatment applied irrigation depths closer to those of the conventional treatment, indicating a more conservative estimation of crop water requirements throughout the growing season. Several factors may explain the lower irrigation depths estimated by the UAV-derived model.

Although the model exhibited satisfactory calibration performance, the use of a single linear relationship may not fully represent temporal changes in crop water requirements, particularly during reproductive stages when NDVI tends to stabilize while evapotranspiration remains high [13,15]. This behavior is consistent with the well-documented phenomenon of NDVI saturation under dense crop canopies, where increases in leaf area index and biomass result in progressively smaller changes in NDVI, thereby reducing its sensitivity to discriminate differences in crop water requirements when maize evapotranspiration remains high [30,31]. Consequently, the model may underestimate K_c_ during periods of maximum canopy development. To overcome this limitation, alternative approaches incorporating red-edge vegetation indices [32,33], thermal information or multi-index models have been shown to improve the estimation of crop water requirements under high biomass conditions [34,35], particularly during late-season stages when NDVI saturation reduces the reliability of NDVI-based approaches [36].

Furthermore, previous studies have been reported that high-resolution remote sensing approaches may underestimate crop water requirements under certain conditions due to limitations associated with spectral band configuration, radiometric calibration, data processing workflows or temporal variability [25,37,38,39]. Likewise, although the UAV-derived K_c_–NDVI model achieved the highest calibration accuracy, its lower operational performance may also reflect the influence of calibration specificity to the 2023 dataset and the interannual variability between calibration and application seasons [40].

An important outcome of this study is that the statistical accuracy of a K_c_–NDVI model does not necessarily translate into superior irrigation scheduling performance. Although the UAV-derived model showed a stronger relationship with lysimeter-based K_c_ values, the corresponding irrigation strategy did not result in improved crop performance. This suggests that model calibration accuracy alone may not be sufficient to evaluate the suitability of remote sensing-based irrigation approaches [41]. The operational performance of remote sensing-based irrigation scheduling depends not only on the strength of the K_c_–NDVI relationship but also on the ability of the model to accurately represent the temporal dynamics of crop water requirements throughout the growing season, particularly during periods of maximum canopy development and evapotranspiration demand [42,43].

In addition to model accuracy, practical implementation should also be considered when selecting a remote sensing platform for irrigation scheduling. Sentinel-2 imagery provides free, regularly available data and broad spatial coverage, making it suitable for large-scale operational applications despite limitations caused by cloud cover [12]. In contrast, UAV imagery offers higher spatial resolution but requires specialized equipment, image processing [14], and trained personnel, increasing operational costs and limiting routine implementation. Therefore, evaluating remote sensing-based irrigation strategies should consider not only predictive performance but also operational feasibility, scalability, and field validation based on agronomic responses.

### 3.3. In-Field Measurements

#### 3.3.1. Stem Diameter

Stem diameter development differed among irrigation scheduling methods. In both hybrids, ID3 generally produced lower stem diameters than ID2, whereas ID2 often exceeded ID1, particularly during intermediate and late growth stages. These results suggest that the lower irrigation depths applied under ID3 imposed moderate water limitations that restricted vegetative growth [44]. Similar responses have been reported in maize, where water deficits reduced stem diameter by approximately 10–23% due to limitations in cell expansion and biomass accumulation [45,46]. These findings suggest that stem diameter is a sensitive indicator of maize response to water availability and can reflect changes in vegetative vigor under deficit irrigation [47].

#### 3.3.2. Plant Height

Plant height was strongly influenced by irrigation management, particularly during the period of rapid vegetative growth. The greater plant height observed under ID2 may be associated with the higher irrigation depths applied compared with ID3. Plant height is highly sensitive to water availability because water deficits reduce cell expansion and leaf elongation [48], processes that depend directly on plant turgor pressure [49,50]. Consequently, the lower irrigation depths applied under ID3 may have limited vegetative growth during periods of high crop water demand, resulting in shorter plants. The absence of significant differences during early growth stages likely reflects the relatively low crop water demand associated with limited canopy development and low transpiration rates during initial vegetative growth [5,51,52]. As canopy expansion accelerated after approximately 55 DAS, differences among irrigation treatments became progressively more evident; indicating that water availability became a stronger determinant of plant growth during periods of rapid canopy development [53,54].

#### 3.3.3. SPAD Chlorophyll Index

SPAD readings followed trends similar to those observed for plant height and stem diameter. The lower values recorded under ID3 are consistent with previous studies showing that chlorophyll content and leaf greenness decline under water deficit conditions [55]. SPAD measurements have been recognized as sensitive indicators of maize physiological status, with reductions commonly associated with decreased chlorophyll synthesis, nutrient uptake, and photosynthetic activity under limited water availability [47,54,56].

#### 3.3.4. In Situ NDVI

In situ NDVI measurements obtained with the GreenSeeker sensor also showed lower values under ID3, particularly during early and intermediate growth stages. Previous studies have shown that reduced irrigation depths affected canopy development and vegetation vigor during periods of rapid crop growth [57,58]. The absence of significant differences during later growth stages may be attributed to NDVI saturation under dense maize canopies, which reduces its sensitivity to changes in biomass, chlorophyll content, and plant physiological status [53,59,60]. This limitation may explain why the ID3, despite its high accuracy, resulted in lower irrigation depths and reduced crop growth, highlighting the need for caution when applying UAV-derived NDVI for irrigation scheduling at advanced growth stages.

### 3.4. Yield, Water Productivity and Bromatological Analyses

The forage yields obtained in the present study were comparable to those previously reported under similar environmental conditions, where fresh forage yields ranging from 55 to 80 t ha^−1^ have been documented [61,62]. ID2 produced the highest forage yields, particularly in hybrid N83N5, whereas ID3, which received the lowest irrigation depths, resulted in lower yields. This response suggests that the UAV-based NDVI approach underestimated crop water requirements, leading to water deficits that reduced biomass accumulation. These limitations have been partly attributed to the saturation of NDVI at advanced phenological stages, which limits its ability to detect variations in crop water status [14,63]. Furthermore, sensor-dependent factors, including spectral band configuration, radiometric calibration, and data processing workflows, are also likely to have influenced model performance [17,26,27,28].

WP highlighted the trade-off between maximizing forage yield and improving irrigation water use efficiency [64,65]. Although ID2 achieved the greatest forage yield, particularly in hybrid N83N5, WP remained comparably high under ID3 owing to the substantial reduction in irrigation water applied. Likewise, in the Matador hybrid, ID3 produced the highest WP among the evaluated irrigation strategies, supporting that moderate reductions in irrigation inputs can enhance water use efficiency even when total forage production declines [66,67]. The present findings are consistent with previous studies in maize reporting that moderate deficit irrigation can improve WP by reducing irrigation inputs proportionally more than biomass production [68,69]. Nevertheless, when water deficits become excessive, the resulting limitations on crop growth and assimilate partitioning may offset these gains by reducing forage yield and nutritional quality [70].

High-quality corn silage is typically characterized by dry matter contents of 30–40%, crude protein concentrations of 6.5–10%, NDF values of 36–48%, ADF values of 18–26%, lignin contents of 2–4%, and starch concentrations of 25–40% of dry matter [71,72,73]. In the present study, irrigation management clearly influenced forage nutritional quality. The lower irrigation depths applied under ID3 resulted in increased NDF, ADF, and lignin concentrations, accompanied by substantial reductions in starch content. These results suggest that water deficits limited grain development and altered biomass partitioning, leading to forage with lower energy value and reduced digestibility [74,75]. In contrast, the N83N5 hybrid under ID2 exhibited the most favorable forage quality profile, characterized by lower fiber concentrations and greater starch accumulation, indicating that adequate irrigation during vegetative growth and grain filling promotes photosynthetic activity, biomass accumulation, and starch deposition in the ears, resulting in greater forage digestibility and energy content [74,76,77].

## 4. Materials and Methods

### 4.1. Description of the Study Area

This study was conducted at the experimental site (25°35′20″ to 25°35′11″ N, and 103°27′02″ to 103°26′53″ W) of the National Center for Disciplinary Research on Water, Soil, Plant, Atmosphere of the National Institute of Forestry, Agricultural and Livestock Research (INIFAP CENID-RASPA), located in Gómez Palacio, Durango, Mexico. The climate in the area is classified as very dry and semi-warm with most precipitation occurring during the summer months. The mean annual temperature ranges from 18 °C to 22 °C, and annual precipitation averages between 100 and 400 mm [78]. The soil is characterized by a predominantly clay loam texture within the first 0.6 m and a loam texture from 0.6 to 1.2 m depth; bulk density is 1.44 g cm^−3^ within the top 0.3 m.

The study was carried out in two phases, corresponding to the spring growing seasons of 2023 (S_1_) and 2024 (S_2_). In the phase S_1_ (from 15 April to 21 July 2023), K_c_–NDVI correlation models were developed. In the phase S_2_ (from 2 April to 6 August 2024), an experiment was conducted to schedule irrigation using data obtained from satellite and UAV imagery, along with the K_c_–NDVI correlation models. Microclimatological data for both study periods, collected by an automated meteorological station (Vantage Pro2TM Plus Wireless, Davis Instruments, Hayward, CA, USA) at the study site, are presented in Table 4.

### 4.2. Development of K_c_–NDVI Models

During phase S_1_, linear regression models were developed to relate the NDVI obtained from satellite and UAV imagery with maize K_c_ values measured using a weighing lysimeter. These empirical relationships were subsequently used to estimate K_c_ values during the phase S_2_. The methodological procedure included: (i) acquisition and processing of Sentinel-2 satellite imagery, (ii) acquisition and processing of UAV imagery, (iii) determination of maize K_c_ using lysimeter measurements, and (iv) development and application of linear regression equations relating NDVI and K_c_.

#### 4.2.1. Satellite NDVI Acquisition

Sentinel-2 is a high-resolution, multispectral Earth observation mission developed by the European Space Agency (ESA) as part of the Copernicus Programme, aimed at monitoring land and vegetation dynamics. It comprises a constellation of two satellites, Sentinel-2A and Sentinel-2B, which together provide a revisit time of five days at the study site. Each satellite carries a Multispectral Instrument capable of capturing imagery in 13 spectral bands ranging from the visible and near-infrared to shortwave infrared, with spatial resolutions of 10, 20, and 60 m [79].

For this study, the red (R) and near-infrared (NIR) bands, corresponding to B4 and B8 bands, respectively, both with a spatial resolution of 10 m, were freely downloaded at level 2A through the Copernicus Open Access Hub (https://browser.dataspace.copernicus.eu/, last accessed on 6 August 2024). Sentinel-2 Level-2A images are atmospherically corrected surface reflectance products derived from Level-1C top-of-atmosphere data. They provide high-resolution multispectral observations corrected for the effects of atmospheric gases, aerosols, and terrain, enabling more accurate analysis of surface properties [79].

The NDVI was calculated using QGIS Software with the following expression:

(1) $${\mathrm{NDVI}}_{\mathrm{satellite}}\;=\;\frac{{\mathrm{NIR}}_{842\;}-\;R_{665}}{{\mathrm{NIR}}_{842}\;+\;R_{665}}$$

where NIR_842_ and R_665_ are the spectral radiance (or reflectance) measurements recorded with sensors in near-infrared (842 nm) and red (665 nm) regions, respectively [80].

The remote sensing data were filtered based on a cloud cover percentage below 30% [81]. For the phase S_1_, the imagery data were acquired across the entire experimental site on the following dates in 2023: April 17 and 27; May 2, 12, 17, and 22; June 1, 6, 11, 16, 21, and 26; and July 11, 16, and 21. For the phase S2, the satellite image data were obtained in the area corresponding to 2 pixels of 10 m × 10 m, previously georeferenced in the study area on the following dates in 2024: April 11, 16, 21 and 26; May 1, 6, 11, 16, 21, 26 and 31; June 5, 10, 15, 25 and 30; and July 5, 10, 15 and 20.

#### 4.2.2. UAV NDVI Acquisition

UAV data collection was performed using a SenseFly eBee Classic (AgEagle Aerial Systems Inc., Raleigh, NC, USA). The UAV was equipped with integrated artificial intelligence to analyze data via its inertial measurement unit and GPS, as well as a 2.4 GHz radio link that enables communication with the flight planning software eMotion 3.5.0 (AgEagle Aerial Systems Inc., Raleigh, NC, USA) through a ground modem with a USB connection. A Parrot Sequoia+ camera (Parrot Drones SAS, Paris, France) with four 1.2 MP multispectral sensors was used to capture the following bands: green (550 nm ± 40 nm), red (660 nm ± 40 nm), red edge (735 nm ± 10 nm), near-infrared (790 nm ± 40 nm), all with global shutters. Additionally, the system included a 16 MP RGB sensor and a sunlight sensor. Radiometric calibration was performed using the empirical line method with a reflectance calibration panel. The flight altitude was set at 116.7 m above ground level. The spatial resolution was 11 cm/pixel, with 70% lateral and 60% longitudinal overlap. The processed images were subsequently rectified, mosaicked, and georeferenced using Pix4D 4.8.4 software (Pix4D S.A., Lausanne, Switzerland). Finally, the NDVI was calculated using QGIS 3.28.3 software, applying the following expression:

(2) $${\mathrm{NDVI}}_{\mathrm{UAV}}=\frac{{\mathrm{NIR}}_{790}\;-\;R_{660}}{{\mathrm{NIR}}_{790}+R_{660}}$$

where NIR_790_ is the reflectance of near-infrared light (790 nm) and R_660_ is the reflectance of red light (660 nm).

UAV imagery for the phase S_1_ was acquired on the following dates in 2023: 15, 17, 22, and 27 April; 2, 8, and 19 May; 1, 7, 22, and 27 June; and 2, 8, 19, and 25 July. For the phase S2, imagery was acquired in 2024 on the following dates: 11, 16, 22, and 29 April; 2, 6, 10, 16, 21, 27, and 31 May; 5, 14, 21, and 27 June; and 1, 5, 10 and 15 July.

#### 4.2.3. Crop Coefficient (K_c_) Determination

The K_c_ of fodder maize is the ratio of the ET_c_ to the ET_0_ and can be calculated by single crop coefficient method [5], using the following two-step approach equation:

(3) $$K_{C}\;=\;\frac{{\mathrm{ET}}_{C}}{{\mathrm{ET}}_{0}}$$

where ET_c_ is the crop evapotranspiration estimated from water balance measurements in lysimeter under fully irrigated conditions (mm day^−1^), and ET_0_ is the evapotranspiration of a reference crop (grass 0.12 m tall) computed from weather data (mm day^−1^).

The ET_c_ of fodder maize was obtained in phase S1, using a weighing lysimeter, which consisted of an undisturbed soil monolith measuring 1.8 m × 3.6 m in surface area and 1.3 m in depth. The surface area of the lysimeter accommodates the common row spacing utilized in the region. An electronic load cell in the weighing system digitally recorded water weight variations from the permanent wilting point (PWP) to field capacity (FC). The weighing equipment was programmed to take and record weight readings every hour to calculate daily evapotranspiration of the crop under fully irrigated conditions (ET_c_). Its accuracy was 0.01 mm, the calibration of lysimeter was conducted at the beginning of the maize growing season so the precision can be ensured. It was located in the 1.8 ha field covered by irrigated maize.

The hybrid maize (N83N5^®^, Syngenta, Mexico City, Mexico) was sown on 15 April 2023 and harvested on 21 July 2023. The planting density was 8 plants per meter, with a row spacing of 0.75 m and a plant spacing of 0.1 m. Furrow irrigation using gated pipe was selected to ensure that the crop was grown under optimal soil moisture conditions, maintaining lysimeter soil water tension between 0.051 and 0.101 MPa, as monitored by a Time Domain Reflectometry (TDR) soil moisture sensor (TRIME^®^-PICO T3/IPH44, IMCO, Ettlingen, Germany). Recommended nutrient rates for regional soils were applied as nitrogen (N) and phosphorus (P) at 100 and 50 kg ha^−1^, respectively. To maintain a similar environment, the same plant type and density were used in the fields surrounding the lysimeter, following identical cultivation practices, except that hand-cultural methods were applied within the lysimeter area.

The ET_0_ was obtained from data collected by an automated meteorological station (Vantage Pro2TM Plus Wireless, Davis Instruments, Hayward, CA, USA) installed at the study site, using the FAO Penman–Monteith method, as follows:

(4) $${\mathrm{ET}}_{0}\;=\frac{\left[0.148∆\left(\mathrm{Rn}\;-\;G\right)+\left(\frac{900_{\gamma }}{T=273}\right)U_{2}\left(e_{s}\;-{\;e}_{a}\right)\right]}{∆\;+\;\gamma (1+0.34U_{2})}$$

where Rn is the net radiation at the crop canopy (MJ m^−2^ per day), G is the soil heat flux (MJ m^−2^ per day), T is the average air temperature (°C), U_2_ is the mean wind speed at 2 m height (m s^−1^), es is saturated vapor pressure deficit, ea is the mean actual vapor pressure deficit (kPa), Δ is the slope of the vapor pressure–temperature curve (kPa °C^−1^), γ is the psychrometric constant (kPa °C^−1^), 0.148 is a coefficient (m^2^∙mm MJ^−1^), and 900 is the conversion factor.

The Kc equation was derived as a function of cumulative crop heat units (CHU), using lysimeter-measured maize evapotranspiration and ET_0_ estimated with the FAO Penman–Monteith method. The use of cumulative CHU to estimate Kc curves has been used in several studies [82,83]. The growing degree days (GDD) method was used to determine CHU as a cumulative temperature during the growing season [84,85] and is expressed as follows.

(5) $$\mathrm{CHU}=\sum_{i=1}^{n}\frac{\mathrm{Tmax}+\mathrm{Tmin}}{2}\;-\;\mathrm{Tbase}$$

where CHU = crop heat unit (°C), Tmax = maximum air temperature (°C), Tmin = minimum air temperature (°C), Tbase = base temperature threshold for maize (10 °C), and n = number of days. The maximum and minimum temperature thresholds of 30 °C and 10 °C, respectively, were used. All temperature values exceeding the threshold were reduced to 30 °C, and values below 10 °C were taken as 10 °C because no growth occurs above or below the threshold (base) temperature values. If the average daily temperature was below the base temperature, the TU value was assumed to be zero [83].

Next, experimental K_c_ was plotted against CHU values to obtain a polynomial equation [86], expressed as follows:

(6) $$\mathrm{Kc}=b_{0}+b_{1}\sum \mathrm{CHU}+b_{2}\sum {\mathrm{CHU}}^{2}+\text{…}+b_{m}\sum {\mathrm{CHU}}^{m}$$

where b_0_ to b_m_ are regression parameters, CHU are crop heat units. The criterion to select the polynomial grade was the coefficient of determination (R^2^).

#### 4.2.4. Development of K_c_–NDVI Linear Regression Models

Linear regression analysis was performed between NDVI and K_c_ to model K_c_–NDVI relationships for each remote sensor [12,13]. Accordingly, two regression models were developed based on either satellite imagery (K_c_–NDVI_Satellite_ model) or UAV imagery (K_c_–NDVI_UAV_ model). For each image acquisition date, the corresponding K_c_ value was used to establish the K_c_–NDVI relationship, ensuring that both variables represented the same stage of crop development [19]. The linear relationship between NDVI and K_c_ was established following the general form:

(7) $$K_{c}\;=\;a(\mathrm{NDVI})\;+\;b$$

where a represents the slope and b the intercept of the regression model.

Model goodness-of-fit was evaluated using the coefficient of determination (R^2^), root mean square error (RMSE), mean absolute error (MAE), and mean bias error (MBE), calculated according to Legates and McCabe [87], and Willmott and Matsuura [88]. These statistics were calculated from the calibration dataset, which consisted of 15 paired observations (*n* = 15) corresponding to the image acquisition dates.

### 4.3. Crop and Irrigation Treatments

In phase S_2_, a field experiment was conducted to apply irrigation to fodder maize crops in near-real time, based on the estimation of ET_c_ using remote sensing data. Two hybrid maize varieties were sown on 2 April 2024, and harvested on 6 August 2024: N83N5^®^ (Syngenta, Mexico City, Mexico) and Matador^®^ (Semillas WOW, Guadalajara, Jalisco, Mexico). The planting density was 8 plants per meter, with a row spacing of 0.75 m and an intra-row (plant-to-plant) spacing of 0.10 m. The irrigation system used was a subsurface drip irrigation system with tape (TORO^®^, Bloomington, MN, USA), caliber 8000, featuring an internal diameter of 16 mm (7/8”), a flow rate of 0.5 L h^−1^ at 15 PSI, and emitters spaced every 10 cm. The tape was buried at a depth of 30 cm.

Treatments were arranged in a split-plot design within a randomized complete block design (RCBD) with three replications. Each plot measured 10 m in length and was six rows wide, with a row spacing of 0.76 m. The two maize hybrids (N83N5 and Matador) were assigned to the main plots, while the three methods used to calculate irrigation depths (ID) were assigned to the subplots. The first method (ID1) corresponded to the irrigation depth traditionally applied by producers in the region. In the second method (ID2), the K_c_ was estimated using the K_c_–NDVI_Satellite_ model. In the third method (ID3), K_c_ was estimated using the K_c_–NDVI_UAV_ model. Irrigation was scheduled based on replacing the water lost due to ET_c_ which was estimated using the following equation [5]:

(8) $${\mathrm{ET}}_{c}={\mathrm{ET}}_{0}*K_{c}$$

where ET_c_ is the crop evapotranspiration (mm day^−1^); ET_0_ is the reference evapotranspiration (mm day^−1^), obtained from an atmometer based on a grass reference crop 0.12 m in height; and, K_c_ the crop coefficient, derived either from satellite imagery (K_c_–NDVI_Satellite_ model) or UAV imagery (K_c_–NDVI_UAV_ model). An allowable depletion of 60% of the available water content was considered during the early phenological stages, and 40% from stage V6 onward. Since Sentinel-2 and UAV imagery were not acquired on a daily basis, the K_c_ value obtained from each image acquisition date was assumed to remain unchanged until the subsequent image acquisition. Daily ET_c_ values were estimated using daily ET_0_ observations and the most recently available K_c_ value for irrigation scheduling purposes.

To calculate the initial irrigation depth (ID_0_), Equation (9) [89] was used, taking into account the soil physical characteristics presented in Table 5.

(9) $${\mathrm{ID}}_{0}=\frac{\left(\theta_{\mathrm{FC}}\;-\;\theta_{\mathrm{PWP}}\right)}{100}*\mathrm{Bd}*z$$

where ID_0_ is the irrigation depth to be applied at the initial irrigation event to restore the soil moisture to field capacity (cm); θ_FC_ is the soil moisture content at field capacity (upper point, %); θ_PWP_ is the soil moisture content at permanent wilting point (lower limit,%); Bd is the bulk density of the soil (g cm^−3^); and z is the depth of plant root zone (cm).

### 4.4. Agronomic Traits and Yield

#### 4.4.1. Plant Sampling

For each 10 × 4.5 m plot, four plants were randomly selected, and their average value was used as the mean for repeated measurements. Sampling was conducted on April 26; May 2, 6, 10, 16, 21, 27, and 31; June 5, 10, 21, and 25; and July 1, 5, 11, 15, 20, and 25. During each sampling event, stem diameter, plant height, SPAD chlorophyll index, and in situ NDVI measured with a GreenSeeker sensor were recorded. Yield and quality forage were determined at the end of the growing season. The selection of sampling dates was primarily determined by satellite overpass frequency (Sentinel-2) and UAV flight campaigns, although some dates were excluded due to cloud cover or precipitation events. Measurements were performed according the following procedures.

#### 4.4.2. Stem Diameter

Stem diameter was measured at the midpoint of the stem [90], with a digital vernier caliper (Mitutoyo, model 500-196-30, Mitutoyo Corp., Kawasaki, Kanagawa, Japan).

#### 4.4.3. Plant Height

Plant height was measured from the base of the stem (soil surface) to the tip of the highest point of the plant. Depending on the growth stage, this highest point corresponded to the tip of the youngest leaf (in vegetative stages) or the tip of the tassel (in later stages) [91,92]. Measurements were taken using a meter ruler or a stadia rod (Topcon, TP-15, Topcon Corp., Tokyo, Japan). To reduce measurement error, each plant was measured three times and the mean value was used as its height.

#### 4.4.4. SPAD Chlorophyll Index

SPAD readings were measured from four randomly selected plants from each plot with a SPAD-502 Chlorophyll meter (Konica Minolta, Osaka, Japan), under consistent lighting conditions in the early morning. Three readings per leaf (one value around the midpoint of the leaf blade and two values 3 cm apart from the midpoint) were averaged as the mean reading for the leaf [93,94].

#### 4.4.5. In Situ NDVI

In situ NDVI was measured using a GreenSeeker^®^ Handheld Crop Sensor (Trimble, Model 505, Sunnyvale, CA, USA). Measurements were taken under consistent light conditions (early morning). For each plot, two central transects were performed with the sensor held at a constant height of 0.60 m above the canopy using a guide. Each transect consisted of a continuous scan (~30 s) and all readings displayed by the device were recorded. The plot NDVI was computed as the mean of all valid readings (outliers due to soil background or obstacles were removed). The procedure follows the GreenSeeker user guide [95] and CIMMYT recommendations [96].

#### 4.4.6. Fodder Maize Yield

The crop was harvested at 120 days after sowing (DAS). Fresh fodder yield was determined by harvesting one randomly placed 1 m^2^ quadrat within each experimental subplot. Plants within the quadrat were cut at ground level and weighed using a field balance to determine fresh weight (FW). Fresh fodder yield was expressed as tons per hectare (t ha^−1^) by extrapolating the biomass collected from the sampled area. Each experimental subplot constituted one experimental unit, resulting in three independent replicates per treatment corresponding to the three blocks.

#### 4.4.7. Water Productivity

Water productivity (WP, kg m^−3^) was calculated as the ratio between crop yield (kg ha^−1^) and total irrigation water applied (m^3^ ha^−1^), following the approach described by Howell [64].

#### 4.4.8. Forage Quality Parameters

At physiological maturity, representative forage samples from each experimental unit were collected for bromatological analysis. Dry matter (DM), crude protein (CP), and starch were determined according to the official methods of AOAC International [97], whereas neutral detergent fiber (NDF), acid detergent fiber (ADF), and acid detergent lignin (ADL) were analyzed following the detergent fiber methodology described by Van Soest et al. [98].

### 4.5. Data Analysis and Statistical Analysis

The experiment was established under a randomized complete block design with a split-plot arrangement and three replications. Main plots corresponded to two maize hybrids (N83N5 and Matador), while subplots were assigned to three irrigation management strategies (ID1, ID2 and ID3).

Stem diameter, plant height, SPAD chlorophyll index, and in situ NDVI, evaluated at 16 dates throughout the crop cycle, were analyzed using linear mixed-effects models for repeated measures, which are appropriate for longitudinal experiments with correlated observations collected from the same experimental units over time [99]. In these models, the fixed effects included hybrid, irrigation treatment, time, and their interactions, whereas blocks and main plots (hybrids) within blocks were treated as random effects, in accordance with the hierarchical structure of the split-plot design [100,101]. To properly model the correlation among repeated measurements within the same experimental unit over time, a continuous first-order autoregressive [CAR(1)] correlation structure was incorporated. This structure accounts for the decreasing correlation between observations as the time lag increases, and is particularly suitable for unequally spaced temporal data [99]. Time was included as a continuous covariate expressed as days after sowing (DAS), and nonlinear temporal responses were modeled using natural cubic splines with three degrees of freedom. The selection of the covariance structure was based on information criteria, including the Akaike Information Criterion (AIC) and the Bayesian Information Criterion (BIC), favoring parsimonious models with adequate fit [102,103].

Statistical analyses were performed in the R environment [104], using the “nlme” package for fitting mixed-effects models with covariance structures [105,106], together with “emmeans” for estimated marginal means and multiple comparisons among treatments [107]. The general model structure was:

(10) $$Y_{\mathrm{ijkl}}\;=\;\mu \;+\;B_{i}\;+\;H_{j}\;+\;R_{k}\;+{\;T}_{l}\;+\;{(H\;\times \;R)}_{\mathrm{jk}}{+\;(H\;\times \;T)}_{\mathrm{jl}}+\;{(R\;\times \;T)}_{\mathrm{kL}}+\;{(H\;\times \;R\;\times \;T)}_{\mathrm{jkl}}+{\;\varepsilon }_{\mathrm{ijkl}}$$

where Y_ijkl_ is the observed response, µ is the overall mean, B_i_ is the random effect of block, H_j_ is the fixed effect of hybrid, R_k_ is the fixed effect of irrigation treatment, T_l_ is the fixed effect of time, and ɛ_ijkl_ represents the residual error with a CAR(1) covariance structure.

Models with and without temporal correlation structures were compared using likelihood ratio tests fitted by maximum likelihood (ML), whereas final parameter estimates were obtained using restricted maximum likelihood (REML), following recommendations for mixed-effects modeling [99,108,109,110]. Model assumptions were evaluated through residual diagnostics, including inspection of quantile–quantile (Q–Q) plots of normalized residuals [111]. When significant effects were detected, estimated marginal means were compared using Tukey-adjusted pairwise tests at a significance level of *p* ≤ 0.05 using the emmeans package (version 2.0.3) [107].

The yield and bromatological variables were analyzed using a linear mixed-effects model appropriate for a split-plot randomized complete block design, considering the effects of hybrid, irrigation, and their interaction. Hybrid, irrigation treatment, and their interaction were considered fixed effects, whereas block and the block × hybrid interaction were included as random effects. Each experimental subplot constituted one experimental unit. When significant effects were detected, mean comparisons were performed using Tukey’s test (*p* ≤ 0.05), controlling the Type I error rate in multiple comparisons [112].

## 5. Conclusions

Both satellite- and UAV-derived NDVI models exhibited strong relationships with lysimeter-derived K_c_ values, confirming the potential of remote sensing for crop coefficient estimation. However, this study demonstrates that model calibration accuracy alone is not sufficient to evaluate the operational performance of remote sensing-based irrigation scheduling. Although the UAV-based model achieved the highest coefficient of determination (R^2^ = 0.9414) and improved water productivity through reduced irrigation inputs, it also induced greater crop water stress, resulting in reduced stem diameter, plant height, chlorophyll status, forage yield, and nutritional quality. In contrast, the satellite-based model provided irrigation depths that more closely matched seasonal crop water requirements, promoting superior crop growth, the highest forage yield, and improved forage quality while maintaining high water productivity. These findings highlight the importance of complementing statistical model evaluation with agronomic validation under field conditions when developing remote sensing-based irrigation strategies. Under the conditions of this study, irrigation scheduling based on Sentinel-2-derived K_c_–NDVI model generally provided the most reliable balance between water use and forage productivity and quality. Nevertheless, these findings should not be generalized to all production systems, as the relative performance of Sentinel-2 and UAV-based approaches may depend on crop phenology, local calibration procedures, sensor characteristics, environmental conditions, irrigation system characteristics, and the potential saturation of NDVI under dense canopies. Therefore, additional validation using independent datasets collected under different climatic conditions, management practices, and growing seasons is required before broader application can be recommended. Future research should explore alternative vegetation indices and multi-sensor approaches to improve the estimation of crop water requirements, particularly during periods of maximum canopy development.

## Figures and Tables

**Table 1:** Cumulative irrigation depth (mm) applied during the crop cycle under the different irrigation management strategies.

Hybrid	ID1 (mm)	ID2 (mm)	ID3 (mm)
N83N5	933	891	717
Matador	933	999	650

ID1: traditional producer irrigation; ID2: satellite-based K_c_ irrigation scheduling; ID3: UAV-based K_c_ irrigation scheduling.

**Table 2:** Analysis of variance of mixed-effects models for maize growth variables under different irrigation management strategies.

Source of Variation	Stem Diameter	Plant Height	SPAD Chlorophyll Index	In Situ NDVI
Hybrid	*F*(1,2) = 0.76; *p* = 0.4765	*F*(1,2) = 1.45; *p* = 0.3521	*F*(1,2) = 0.09; *p* = 0.7981	*F*(1,2) = 2.20; *p* = 0.2765
Irrigation	*F*(2,260) = 24.22; *p* < 0.0001 ***	*F*(2,260) = 8.91; *p* = 0.0002 ***	*F*(2,260) = 23.56; *p* < 0.0001 ***	*F*(6,260) = 6.67; *p* = 0.0015 **
Time	*F*(3,260) = 360.68; *p* < 0.0001 ***	*F*(3,260) = 600.87; *p* < 0.0001 ***	*F*(3,260) = 14.56; *p* < 0.0001 ***	*F*(3,260) = 168.55; *p* ≤ 0.0001 ***
Hybrid × Irrigation	*F*(2,260) = 0.06; *p* = 0.9449	*F*(2,260) = 0.2464; *p* = 0.7818	*F*(2,260) = 0.08; *p* = 0.9247	*F*(2,260) = 1.69; *p* = 0.1864
Hybrid × Time	*F*(3,260) = 1.14; *p* = 0.3353	*F*(3,260) = 3.50; *p* = 0.0162 *	*F*(3,260) = 0.29; *p* = 0.8321	*F*(3,260) = 1.35; *p* = 0.2598
Irrigation × Time	*F*(6,260) = 2.03; *p* = 0.0626	*F*(6,260) = 3.47; *p* = 0.0026 **	*F*(6,260) = 0.49; *p* = 0.8193	*F*(6,260) = 1.56; *p* = 0.1589
Hybrid × Irrigation × Time	*F*(6,260) = 1.27; *p* = 0.2717	*F*(6,260) = 0.17; *p* = 0.9838	*F*(6,260) = 0.08; *p* = 0.9978	*F*(6,260) = 0.50; *p* = 0.8083

Values are presented as *F*(NumDF,DenDF) = *F* statistic; *p* = probability associated with the F test. NumDF, numerator degrees of freedom; DenDF, denominator degrees of freedom. Asterisks indicate significant effects at * *p* ≤ 0.05, ** *p* ≤ 0.01, and *** *p* ≤ 0.001.

**Table 3:** Yield, water productivity and forage quality parameters under three irrigation strategies: traditional producer irrigation (ID1), satellite-based K_c_ irrigation scheduling (ID2), and UAV-based K_c_ irrigation scheduling (ID3).

Hybrid	ID	Yield (t ha^−1^)	WP (kg m^−3^)	DM (%)	CP (%)	NDF (%)	ADF (%)	Lignin (%)	Starch (%)
N83N5	ID1	44.6 bc	4.78 a	35.28 c	10.64 b	41.49 a	22.25 a	4.43 abc	25.74 d
	ID2	59.8 d	6.71 c	40.55 d	8.96 a	37.90 a	19.73 a	4.17 a	37.88 e
	ID3	46.5 c	6.49 bc	27.66 ab	9.05 a	56.22 c	33.44 c	5.60 c	13.54 bc
Matador	ID1	41.9 ab	4.49 a	25.41 a	9.58 a	53.19 bc	30.24 b	4.38 ab	10.36 ab
	ID2	45.8 bc	4.58 a	29.90 b	9.10 a	51.06 b	31.24 bc	5.09 abc	16.87 c
	ID3	39.6 a	6.09 b	24.44 a	9.52 a	56.00 c	34.01 c	5.45 bc	8.30 a

Different lowercase letters indicate significant differences among irrigation treatments according to Tukey’s test (*p* < 0.05). WP, water productivity; DM, dry matter; CP, crude protein; NDF, neutral detergent fiber; ADF, acid detergent fiber.

**Table 4:** Average weather conditions of experimental site during maize growing seasons S_1_ (15 April–21 July 2023) and S_2_ (2 April–6 August 2024).

Weather Variable	Unit	S_1_ Data	S_2_ Data
Precipitation	mm	178.5	116.3
Maximum air temperature	°C	34.1	35.7
Minimum air temperature	°C	18.5	19.5
Vapor pressure	Pa	1528.9	1012.2
Photoperiod	s día^−1^	47,962.9	47,614.5
Solar irradiance	W m^−2^ día^−1^	465.3	489.4

**Table 5:** Soil physical characteristics at the experimental site.

Property	Values
Field capacity (FC)	33.9%
Permanent wilting point (PWP)	19.2%
Bulk density (Bd)	1.44 g cm^−3^
Rooting depth (z)	30 cm

## Data Availability

The data presented in this study are available on request from the corresponding authors.

## Abbreviations

The following abbreviations are used in this manuscript:

UAV	Unmanned aerial vehicle
NDVI	Normalized difference vegetation index
ET_c_	Crop evapotranspiration
ET_0_	Reference evapotranspiration
DAS	Days after sowing
ID	Irrigation depth
CHU	Crop heat units
CAR	Correlation among repeated measurements
SPAD	Soil plant analysis development
FW	Fresh weight
DM	Dry matter
CP	Crude protein
NDF	Neutral detergent fiber
ADF	Acid detergent fiber
GDD	Growing degree days
R	Red
NIR	Near-infrared
ESA	European Space Agency
ANOVA	Analysis of variance
RMSE	Root Mean Squared Error
MAE	Mean Absolute Error
MBE	Mean Bias Error

## Appendix A

### Appendix A.1

**Table A1:** Tukey comparisons for plant height.

Hybrid	DAS	Comparison	Mean Difference	*p*-Value	Significance
Matador	55	ID2 vs. ID3	30.290561	0.0300236	*
Matador	60	ID2 vs. ID3	34.714935	0.00834562	**
Matador	65	ID2 vs. ID3	38.8354122	0.00193858	**
Matador	70	ID2 vs. ID3	42.4824865	0.00050781	***
Matador	75	ID2 vs. ID3	45.4866515	0.00020797	***
Matador	80	ID2 vs. ID3	47.6784009	0.00014558	***
Matador	85	ID2 vs. ID3	48.8882283	0.00014586	***
Matador	90	ID2 vs. ID3	48.9466276	0.00016438	***
Matador	95	ID1 vs. ID2	−27.8363454	0.04813807	*
Matador	95	ID2 vs. ID3	47.7226429	0.00018599	***
Matador	100	ID1 vs. ID2	−28.2943648	0.03610059	*
Matador	100	ID2 vs. ID3	45.3702308	0.00025831	***
Matador	105	ID1 vs. ID2	−28.5927235	0.03130905	*
Matador	105	ID2 vs. ID3	42.1710463	0.00064946	***
Matador	110	ID1 vs. ID2	−28.7846416	0.03855379	*
Matador	110	ID2 vs. ID3	38.4073469	0.00334987	**
Matador	115	ID2 vs. ID3	34.3613901	0.02167842	*
N83N5	55	ID2 vs. ID3	28.379577	0.04565632	*
N83N5	60	ID2 vs. ID3	32.5683902	0.01457048	*
N83N5	65	ID1 vs. ID3	29.7771161	0.0238573	*
N83N5	65	ID2 vs. ID3	36.1933916	0.00428012	**
N83N5	70	ID1 vs. ID3	31.6278097	0.01359772	*
N83N5	70	ID2 vs. ID3	39.2009949	0.00149681	**
N83N5	75	ID1 vs. ID3	32.7451398	0.01099561	*
N83N5	75	ID2 vs. ID3	41.5376136	0.0007999	***
N83N5	80	ID1 vs. ID3	33.0828055	0.01252254	*
N83N5	80	ID2 vs. ID3	43.1496613	0.00067103	***
N83N5	85	ID1 vs. ID3	32.5945061	0.01738124	*
N83N5	85	ID2 vs. ID3	43.9835514	0.00072964	***
N83N5	90	ID1 vs. ID3	31.2339409	0.02549726	*
N83N5	90	ID2 vs. ID3	43.9856976	0.00081779	***
N83N5	95	ID1 vs. ID3	28.9752548	0.03758415	*
N83N5	95	ID2 vs. ID3	43.1230157	0.00084039	***
N83N5	100	ID2 vs. ID3	41.5139478	0.00093585	***
N83N5	105	ID2 vs. ID3	39.34485	0.00161761	**
N83N5	110	ID2 vs. ID3	36.8023991	0.0052555	**
N83N5	115	ID2 vs. ID3	34.0732714	0.02307253	*

Asterisks indicate significant differences among irrigation treatments at each sampling date according to Tukey’s multiple comparison test: * *p* ≤ 0.05, ** *p* ≤ 0.01, and *** *p* ≤ 0.001.

### Appendix A.2

**Table A2:** Tukey comparisons for stem diameter.

Hybrid	DAS	Comparison	Mean Difference	*p*-Value	Significance
Matador	60	ID2 vs. ID3	0.38498711	0.01928725	*
Matador	65	ID1 vs. ID2	−0.39028785	0.01057289	*
Matador	65	ID2 vs. ID3	0.42080586	0.0051802	**
Matador	70	ID1 vs. ID2	−0.44324907	0.00214721	**
Matador	70	ID2 vs. ID3	0.45112829	0.001738	**
Matador	75	ID1 vs. ID2	−0.48820241	0.00087194	***
Matador	75	ID2 vs. ID3	0.47791482	0.00115525	**
Matador	80	ID1 vs. ID2	−0.52239851	0.00077676	***
Matador	80	ID2 vs. ID3	0.50312585	0.00127682	**
Matador	85	ID1 vs. ID2	−0.54308803	0.00092802	***
Matador	85	ID2 vs. ID3	0.52872178	0.0013161	**
Matador	90	ID1 vs. ID2	−0.54752161	0.00095641	***
Matador	90	ID2 vs. ID3	0.556663	0.00076475	***
Matador	95	ID1 vs. ID2	−0.53353216	0.00071297	***
Matador	95	ID2 vs. ID3	0.58865913	0.00016045	***
Matador	100	ID1 vs. ID2	−0.50325584	0.00060731	***
Matador	100	ID2 vs. ID3	0.62456617	1.44 × 10^−5^	***
Matador	105	ID1 vs. ID2	−0.46075755	0.00188777	**
Matador	105	ID2 vs. ID3	0.66340932	3.68 × 10^−6^	***
Matador	110	ID1 vs. ID2	−0.41011127	0.02124187	*
Matador	110	ID2 vs. ID3	0.70420987	1.93 × 10^−5^	***
Matador	115	ID2 vs. ID3	0.74598914	0.0003457	***
N83N5	35	ID1 vs. ID2	−0.32781186	0.03880278	*
N83N5	40	ID1 vs. ID2	−0.33297802	0.0338397	*
N83N5	40	ID2 vs. ID3	0.38440483	0.01135292	*
N83N5	45	ID2 vs. ID3	0.45530942	0.0042819	**
N83N5	50	ID2 vs. ID3	0.5070972	0.00230883	**
N83N5	55	ID2 vs. ID3	0.5364994	0.00108941	**
N83N5	60	ID2 vs. ID3	0.54628906	0.00043084	***
N83N5	65	ID2 vs. ID3	0.54074969	0.00020179	***
N83N5	70	ID2 vs. ID3	0.52416478	0.00021105	***
N83N5	75	ID2 vs. ID3	0.50081781	0.00061328	***
N83N5	80	ID2 vs. ID3	0.47499229	0.00256318	**
N83N5	85	ID2 vs. ID3	0.45097171	0.00757581	**
N83N5	90	ID2 vs. ID3	0.43303955	0.01199081	*
N83N5	95	ID2 vs. ID3	0.42491417	0.00931875	**
N83N5	100	ID1 vs. ID2	−0.34379375	0.02878393	*
N83N5	100	ID2 vs. ID3	0.42613715	0.00459836	**
N83N5	105	ID1 vs. ID2	−0.39424889	0.00962674	**
N83N5	105	ID2 vs. ID3	0.43437802	0.00369844	**
N83N5	110	ID1 vs. ID2	−0.45086021	0.00975465	**
N83N5	110	ID2 vs. ID3	0.44729748	0.0104688	*
N83N5	115	ID1 vs. ID2	−0.5105496	0.02153977	*
N83N5	115	ID2 vs. ID3	0.46255623	0.04220019	*

Asterisks indicate significant differences among irrigation treatments at each sampling date according to Tukey’s multiple comparison test: * *p* ≤ 0.05, ** *p* ≤ 0.01, and *** *p* ≤ 0.001.

### Appendix A.3

**Table A3:** Tukey comparisons for SPAD chlorophyll index.

Hybrid	DAS	Comparison	Mean Difference	*p*-Value	Significance
Matador	60	ID2 vs. ID3	6.7041182	0.03026683	*
Matador	65	ID2 vs. ID3	7.00747599	0.01348502	*
Matador	70	ID2 vs. ID3	7.30644223	0.00707236	**
Matador	75	ID2 vs. ID3	7.59170532	0.00633962	**
Matador	80	ID2 vs. ID3	7.8539537	0.00832354	**
Matador	85	ID2 vs. ID3	8.08387577	0.0103863	*
Matador	90	ID2 vs. ID3	8.27215996	0.00902874	**
Matador	95	ID2 vs. ID3	8.41122814	0.00446154	**
Matador	100	ID2 vs. ID3	8.50631359	0.00162749	**
Matador	105	ID2 vs. ID3	8.56839168	0.00162205	**
Matador	110	ID2 vs. ID3	8.60846485	0.00872074	**
N83N5	35	ID2 vs. ID3	6.3215276	0.02837454	*
N83N5	40	ID2 vs. ID3	6.80042197	0.01458353	*
N83N5	45	ID2 vs. ID3	7.22413213	0.01630037	*
N83N5	50	ID2 vs. ID3	7.57426336	0.01801078	*
N83N5	55	ID2 vs. ID3	7.83807359	0.01351998	*
N83N5	60	ID2 vs. ID3	8.02543141	0.00700372	**
N83N5	65	ID2 vs. ID3	8.15185809	0.00312155	**
N83N5	70	ID2 vs. ID3	8.23287487	0.00196408	**
N83N5	75	ID2 vs. ID3	8.28400303	0.0025077	**
N83N5	80	ID2 vs. ID3	8.32076382	0.00472879	**
N83N5	85	ID2 vs. ID3	8.35867849	0.00765204	**
N83N5	90	ID2 vs. ID3	8.41326831	0.0077191	**
N83N5	95	ID2 vs. ID3	8.49810249	0.0040035	**
N83N5	100	ID1 vs. ID3	6.40776893	0.02443825	*
N83N5	100	ID2 vs. ID3	8.61232339	0.00139473	**
N83N5	105	ID1 vs. ID3	6.58880349	0.02095176	*
N83N5	105	ID2 vs. ID3	8.74860719	0.00124876	**
N83N5	110	ID2 vs. ID3	8.8995996	0.00636294	**
N83N5	115	ID2 vs. ID3	9.05794632	0.03835798	*

Asterisks indicate significant differences among irrigation treatments at each sampling date according to Tukey’s multiple comparison test: * *p* ≤ 0.05, ** *p* ≤ 0.01.

### Appendix A.4

**Table A4:** Tukey comparisons for in situ NDVI.

Hybrid	DAS	Comparison	Mean Difference	*p*-Value	Significance
Matador	35	ID2 vs. ID3	0.13328772	0.00756355	**
Matador	40	ID1 vs. ID2	−0.10740532	0.03192154	*
Matador	40	ID2 vs. ID3	0.15628841	0.00081405	***
Matador	45	ID1 vs. ID2	−0.1194283	0.02784157	*
Matador	45	ID2 vs. ID3	0.17123327	0.00075853	***
Matador	50	ID1 vs. ID2	−0.12299386	0.03811576	*
Matador	50	ID2 vs. ID3	0.17543703	0.00150171	**
Matador	55	ID2 vs. ID3	0.16717911	0.00293925	**
Matador	60	ID2 vs. ID3	0.14859776	0.00624953	**
Matador	65	ID2 vs. ID3	0.12279591	0.01906434	*
N83N5	40	ID1 vs. ID3	0.12916523	0.00722613	**
N83N5	40	ID2 vs. ID3	0.10814724	0.03046834	*
N83N5	45	ID1 vs. ID3	0.14972771	0.00387758	**
N83N5	45	ID2 vs. ID3	0.12216724	0.02368115	*
N83N5	50	ID1 vs. ID3	0.15803589	0.00490539	**
N83N5	50	ID2 vs. ID3	0.12707901	0.03073328	*
N83N5	55	ID1 vs. ID3	0.15215219	0.00774592	**
N83N5	55	ID2 vs. ID3	0.12144272	0.04352963	*
N83N5	60	ID1 vs. ID3	0.13464299	0.0151116	*
N83N5	65	ID1 vs. ID3	0.10920069	0.04286457	*

Asterisks indicate significant differences among irrigation treatments at each sampling date according to Tukey’s multiple comparison test: * *p* ≤ 0.05, ** *p* ≤ 0.01, and *** *p* ≤ 0.001.
